# Emphasis on Early Prenatal Diagnosis and Perinatal Outcomes Analysis of Apert Syndrome

**DOI:** 10.3390/diagnostics14141480

**Published:** 2024-07-10

**Authors:** Valentin Nicolae Varlas, Dragos Epistatu, Roxana Georgiana Varlas

**Affiliations:** 1 Department of Obstetrics and Gynecology, Filantropia Clinical Hospital, 011132 Bucharest, Romania; valentin.varlas@umfcd.ro; 2 Faculty of Dentistry, “Carol Davila” University of Medicine and Pharmacy, 010221 Bucharest, Romania; 3 Department of Radiology, Faculty of Dentistry, “Carol Davila” University of Medicine and Pharmacy, 17-21 Calea Plevnei Street, 020021 Bucharest, Romania; 4 Doctoral School, “Carol Davila” University of Medicine and Pharmacy, 020021 Bucharest, Romania; roxana-georgiana.bors@drd.umfcd.ro

**Keywords:** Apert syndrome, craniosynostosis, early prenatal diagnosis, perinatal outcome, second-trimester screening, prognosis

## Abstract

Apert syndrome is an inherited condition with autosomal dominant transmission. It is also known as acrocephalosyndactyly type I, being characterized by a syndrome of craniosynostosis with abnormal head shape, facial anomalies (median hypoplasia), and limb deformities (syndactyly, rhizomelic shortening). The association can suspect the prenatal diagnosis of these types of anomalies. The methodology consisted of revising the literature, by searching the PubMed/Medline database in which 27 articles were selected and analyzed, comprising 32 cases regarding the prenatal diagnosis of Apert syndrome. A series of ultrasound parameters, the anatomopathological abnormalities found, the obstetric results, and the genetic tests were followed. The distribution of imaging results (US, MRI) identified in the analyzed cases was as follows: skull-shaped abnormalities were evident in 96.8% of cases, facial abnormalities (hypertelorism 43.7%, midface hypoplasia 25%, proptosis 21.8%), syndactyly in 87.5%, and cardiovascular abnormalities in 9.3%. The anomalies detected by the ultrasound examination of the fetus were confirmed postnatally by clinical or gross evaluation or imaging. The management of these cases requires an early diagnosis, an evaluation of the severity of the cases, and appropriate parental counseling.

## 1. Introduction

Apert syndrome is a severe autosomal dominant disorder caused by gene mutations that encode fibroblast growth factor receptor 2 (FGFR2) on chromosome 10q [1,2]. Most cases appear de novo because of new mutations, so familial recurrence risk is low. However, more than 98% of all cases are infrequent and associated with advanced paternal age [3,4]. It is defined by early fusion of the cranial sutures (craniosynostosis) and syndactyly in the hands and feet, other associated anomalies having a low rate of occurrence (skeletal, cerebral, skin, internal organs, and oral/maxillofacial region) [1,5,6]. Apert syndrome is a rare condition, accounting for 6 to 15.5 out of 1 million live births, and it represents 4.5% of all cases diagnosed with craniosynostosis syndromes [7,8]. The condition’s prevalence was 2.9 times higher in Asians compared to Hispanics, and the distribution related to the child’s sex (male–female) varied between 0.79 and 0.94 [9].

Establishing an early prenatal diagnosis, especially of severe forms, is very important for counseling parents regarding the poor prognosis of these cases with serious sequelae, and in the case of mild forms of pregnancy monitoring, planning the moment of birth and their neonatal support [7].

The main effects of craniofacial dysmorphism described in Apert syndrome: brachycephalic skull, cranial asymmetry, prominent forehead, hypertelorism and ocular proptosis, exophthalmos, expansion in the temporal region, premature fusion of the coronal suture, widened metopic suture, ventriculomegaly, agenesis/dysgenesis of the corpus callosum, septal leaflets, and olfactory tract. These changes, depending on the degree of damage, can cause respiratory disorders, breastfeeding difficulty, and possible eye injuries at birth [10]. Besides the anomalies that define the syndrome, the association of cardiovascular anomalies (atrial/ventricular septum defects, patent foramen ovale) was 23.5%, cleft palate 23.5%, urinary (hydronephrosis) 5.9%, and central nervous system (agenesis corpus callosum, gyral anomalies) 5.9% [3].

The 2D prenatal ultrasound evaluation of the fetus can reveal several anomalies characteristic of this syndrome by highlighting facial morphology anomalies (facial hypoplasia, orbital, pharyngeal bone anomalies) and syndactyly (fusion of fingers and/or toes). The 3D imaging reconstruction of the fetal skull and limbs (ultrasound, MRI) is complementary, being crucial in establishing the diagnosis of Apert syndrome and differentiating it from other craniosynostosis [11,12]. Carrying out the 4D ultrasound can reveal the vicious positions that affect fetal mobility. In the absence of the fetal anomalies characteristic of this syndrome, it is necessary to establish the genetic diagnosis by karyotype from the amniotic fluid. Establishing the diagnosis and its severity is important in correct parental counseling.

Often, managing cases with less severe evolution requires a multidisciplinary perspective involving a plastic, oral, maxillofacial, and orthodontic surgical approach, recovery of motor function, and psychological counseling. In cases of Apert syndrome, the medical team requires the involvement of a medical geneticist to establish the FGFR2 mutation; the definitive diagnosis can be confirmed only by molecular testing. In cases of fetal death, the pathologist is necessary to confirm the anomalies detected by imaging and to identify other possible anomalies associated that could not be diagnosed prenatally [10].

This article aims to review the reported cases of severe Apert syndrome detected early prenatally during second-trimester ultrasound screening and to highlight the role of parental counseling, therapeutic management, and prognosis. It has been observed that most cases are diagnosed late in the third trimester when managing these situations becomes much more difficult.

## 2. Literature Review

Apert syndrome (acrocephalosyndactyly type I) is a syndrome with a low incidence rate characterized by the following classic triad: craniosynostosis, flat midface, and bilateral syndactyly [7].

Normal brain development will determine the proper development of the fetal skull. Thus, the process of closing the cranial sutures takes place according to the following model: Initially, the metopic suture is closed at the age of two, and then until adulthood, the other sutures are closed after the completion of craniofacial development. When craniosynostosis is an isolated anomaly, it frequently involves the sagittal suture [13], compared to that which is part of a genetic syndrome (Apert, Pfeiffer, Crouzon, Carpenter, and Saethre–Chotzen), when several sutures are affected. In Apert syndrome, a bicoronal synostosis is observed [14]. The genetic syndromes that associate craniosynostosis determined by FGFR2 gene mutations are autosomal dominant and show a variable degree of overlap of some clinical signs. The exception is Carpenter syndrome, caused by the RAB23 mutation, which is autosomal recessive [15].

Prenatal identification of the presence of craniosynostosis is important because it induces long-term complications such as impairment of neurodevelopment and cognitive function, increased intracranial pressure (Chiari malformation), impairment of cranial nerve function with vision, hearing and speech deficits, and psychological problems. Ultrasound evidence of hydrocephalus is found in approximately 8% of all cases of craniosynostosis; in the case of Apert syndrome, ventriculomegaly is usually non-progressive [16]. The prenatal diagnosis of this syndrome is based on the ultrasonographic examination. There is no specific sign of Apert syndrome in the first trimester due to the insufficient sonographic resolution of the developing fetal structures, such as the fingers. It may be associated with increased nuchal translucency [17].

The severity of cases with Apert syndrome can be evaluated based on the associated craniofacial anomalies that can cause damage to the respiratory tract (reduction in naso/oropharyngeal spaces) and visual impairment. Thus, the prenatal assessment of the severity of craniosynostosis based on the classification of Lu et al. (bicoronal synostosis, pansynostosis, and perpendicular combination of synostosis) can be used as a possible management tool for these patients [18]. Any other element of associated anatomical distortion (e.g., mitten hands, proptosis) increases the degree of severity of these cases, negatively influencing the prognosis [11]. Delahaye et al. demonstrated that suture closure observed in the third trimester of pregnancy was preceded by anatomical distortions and deformations of the fetal skull shape by 4 to 16 weeks [19]. Deformation of the fetal skull shape, abnormal biometry, and ventriculomegaly, identified by 2D/3D ultrasound or MRI, can be imaging markers of fetal craniosynostosis [20,21].

**Figure 1:**
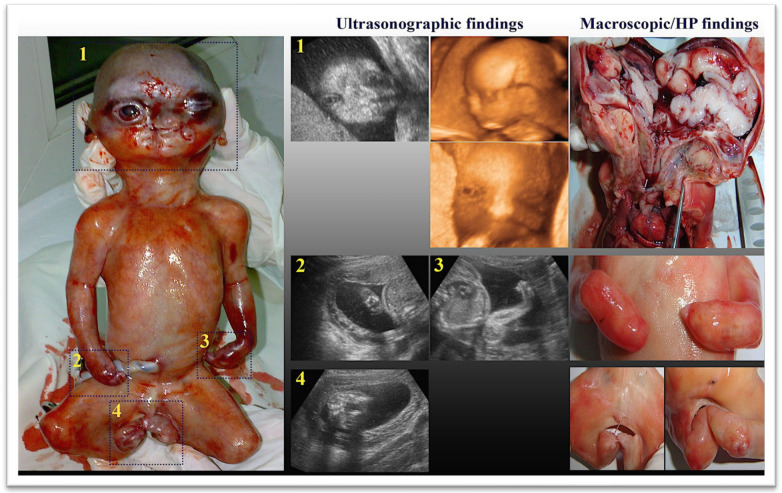
Ultrasonographic and macroscopic findings—fetal cranium abnormalities (acrocephaly and brachycephaly), prominent forehead, nasal bone hypoplasia, facial dysmorphism (flat midface, hypertelorism, exorbitism), cleft lip and palate and low-set ears, hands syndactyly, feet syndactyly type IVa at all fingers, with median fusion of the low extremities, ambiguous external genital organs. A gross examination of the head and brain revealed craniosynostosis, hypertelorism, exorbitism, hypoplastic nose, pharynx abnormality, gyral anomalies, hypoplastic white matter, and heterotopic gray matter (personal collection) [24].

In the second trimester, classic features of Apert syndrome include acrocephaly and brachycephaly, craniofacial dysmorphic elements: flat forehead, maxillary hypoplasia causing a flat midface, hypertelorism, bilateral exophthalmos, low-set ears, small and flat nose, and cleft palate (in one-third of cases). In the conditions of a hypoplastic or malformed pharynx, severe respiratory problems can be associated. During the prenatal examination, possible bone abnormalities can be identified, such as syndactyly present both in the hands and feet, and it is necessary to establish the type of syndactyly (often “mitten-like” hand). Other possible anomalies in the hands and feet that can be identified are the absence of the middle phalanges and absent or supernumerary carpal/tarsal bones [22,23] (Figure 1).

Nonspecific ultrasound findings in Apert syndrome include anomalies of the central nervous system (agenesis of the corpus callosum, ventriculomegaly, gyral abnormalities, posterior fossa malformations), thickened nuchal fold, cardiovascular (atrial and ventricular septal defect, patent foramen ovale), urinary (hydronephrosis, polycystic kidney), genital (cryptorchidia), omphalocele or diaphragmatic hernia, polyhydramnios, and esophageal atresia. These can precede the appearance of skeletal changes [22,23].

Other syndromes associated with syndactyly are facial digital syndrome type I, Cornelia de Lange, Russel Silver, Opitz, Adams Oliver, Holt Oram, Roberts, Fraser, Treacher Collins, and Nager syndromes. They are also associated with other anomalies, such as skeletal defects, but they do not involve the presence of craniosynostosis [25].

Fibroblast growth factor (FGF) is a protein that acts through FGF receptor (FGFR) with tyrosine kinase activity and has implications in morphogenesis, migration, proliferation, and differentiation of stem cells involving osteoblasts and chondroblasts. FGF is responsible for the development of the axial and craniofacial skeletal. The study of the molecular basis of Apert syndrome revealed a genetic mutation of the Ser252Trp or Pro253Arg amino acids, which link the immunoglobulin-like domains II and III of FGFR2. The S252W amino acid mutation is the most frequent and associates severe craniofacial anomalies, and the Pro253Arg amino acid mutation associates severe syndactyly and less severe craniofacial defects [4,17]. FGF8 and FGF10 trigger limb bud development by proliferation of undifferentiated mesenchymal cells. Limb growth results from regulating various genes, including those of the HOX family, and from the effects of FGFs. Mutations in the genes encoding FGFR1, FGFR2, and FGFR3 can cause skeletal dysplasias and craniosynostosis syndromes [26]. The differential diagnosis of Apert syndrome is difficult due to the overlap of clinical signs with other syndromes with craniosynostosis (Table 1).

The early prenatal diagnosis of Apert syndrome and, respectively, of other syndromes involving craniosynostosis is correlated with the prognosis of these cases and the therapeutic management related to the gestational age at which the diagnosis is established; because in many countries, according to the legislation, the termination of pregnancy argued by for medical reasons is allowed up to 24 weeks of gestation. Therefore, we performed an all-time electronic search of the specialized literature. We accessed the PubMed database and selected the articles (case series and case reports) written in English, French, and Spanish. Search terms were “craniosynostosis”, “Apert syndrome”, “early prenatal diagnosis”, “second-trimester screening”, “treatment”, “prognosis”, AND “fetuses” OR “newborns”. In the end, 27 articles were eligible, totaling 32 cases included in this article’s review (Table 2).

The average maternal age in the presented cases was 31.9 (range 23–39). The average gestational age at which changes suggestive of Apert syndrome were identified in the US was 21.3 weeks (range 17.3–24).

Early detection in the second trimester is difficult because the standard anomalies (craniofacial deformities, syndactyly) are often preceded by a non-specific set of anomalies (cardiovascular, central nervous system, renal, digestive) responsible for the heterogeneous nature of the clinical profile. As a result, the severity of Apert forms is assessed based on the early identification of one or more associated anomalies.

Furthermore, 23/32 (71.8%) patients were molecularly tested, of which 82.6% (*n* = 19) of patients opted for termination of pregnancy (TOP). According to the analysis of the presented cases, it is observed that early prenatal detection is accompanied by an increased rate of TOP (75%) due to the severity of the ultrasound features compared to the cases where the ultrasound markers are detected late. As a result, a careful and systematic ultrasound evaluation during the second trimester is crucial for the prognosis of these cases.

In 31/32 (96.8%) of the cases, there were changes in the shape of the head, of which 26/31 (83.7%) were visualized by ultrasound and 5 by subsequent MRI scanning. In one case, the second-trimester scan showed a normal shape of the skull, and a re-evaluation performed after 3 weeks showed colpocephaly and, after another 3 weeks, craniosynostosis and widely open metopic suture. Thus, two cases of agenesis of the corpus callosum (ACC) were suspected; the MRI analysis confirmed it only in one case. Also, an MRI was used to establish the diagnosis in a case of corpus callosum dysgenesis. Hypertelorism in 14/32 (43.7%) was visualized sonographically in 78.5% (*n* = 11) cases, and three cases were detected after MRI scanning. The diagnosis of proptosis was established sonographically in 6 out of 11 cases, one was identified after performing an MRI, and 4 cases (36.3%) were diagnosed during fetal examination after TOP.

Anomalies of the fingers (syndactyly) were diagnosed by prenatal imaging in 28 cases (87.5%), of which 18 cases (60%) involved the hands and the feet. The syndactyly involving only the hands was observed antenatally in 10 cases, with mitten type in 9 cases. Additionally, postmortem fetal examination revealed 3 cases of mitten hands.

Antenatal imaging evaluation also identified three cases of cardiovascular anomalies (hypoplastic left ventricle and ascending aorta, coarctation of the aorta, ventricular septal defect, and overriding of the aorta), two cases of renal anomalies (bilateral hydronephrosis), one case with abnormally expanded lungs confirmed both sonographically and MRI, and one case of digestive anomaly (omphalocele) (Table 3).

The treatment of patients with Apert syndrome is a real challenge, requiring a multidisciplinary team and scheduled surgical corrections at different stages of development. Thus, craniosynostosis and disorders secondary to brain compression are repaired by craniotomy in the first year of life, palate repair between 6 and 14 months, syndactyly between 1 and 4 years, jaw surgery, and mid-face anomalies between 4 and 6 years, after the completion of growth orthognathic correction. All other associated manifestations, such as strabismus, hearing loss, and sleep apnea, require surgical or drug treatment, depending on severity, to improve the patient’s quality of life [57,58,59].

The prognosis, social integration, and quality of life of these patients depend on the severity of the condition. Some patients, although psychologically supported by the family, even after the therapeutic interventions, may present intellectual disabilities or psychosocial disorders.

## 3. Conclusions

The presence of suggestive ultrasound changes and/or associated anomalies makes early prenatal diagnosis of Apert syndrome an important tool in the optimal assessment of the degree of severity for each case. The use of 3D ultrasound allowed a better understanding by the parents of the fetal damage, facilitating counseling and providing useful information regarding the decision to terminate the pregnancy in cases with an unfavorable prognosis due to long-term complications such as impaired neurologic and cognitive development or problems caused by facial dysmorphism, craniosynostosis, and skeletal malformations.

## Figures and Tables

**Table 1:** Differential diagnosis of Apert syndrome.

Syndrome	Incidence	Inheritance	Gene Mutations	Common Anomalies	Associated Anomalies	**Outcome**
Apert [7,8]	6–15.5 in 1 million live births	Autosomal dominant	FGFR2 on chromosome 10q	Craniosynostosis, midface hypoplasia, and syndactyly in the hands and feet	Cardiovascular anomalies (atrial/ventricular septum defects, patent foramen ovale), cleft palate, central nervous system (agenesis corpus callosum, gyral anomalies), and urinary (hydronephrosis)	Cognitive impairment, respiratory complications, neurologic deficits, ocular anomalies
Crouzon [27] Crouzon + acanthosis nigricans [28,29]	16 in 1 million live births	Autosomal dominant	FGFR2	Compared to Apert, the degree of facial dysmorphism is milder, and the presence of cleft palate is rare. They usually have normal hands and feet	Cervical spine malformations	Strabismus, amblyopia, sleep apnea. Cognitive disabilities according to the severity of the cranial abnormality
	1 in 1 million live births	Autosomal dominant	FGFR3	Craniosynostosis, midface hypoplasia, choanal atresia	Narrow sciatic notch, short vertebra, and broad metacarpals	
Pfeiffer [30]	10 in 1 million live births	Autosomal dominant	FGFR1/FGFR2	Compared to Apert, the degree of syndactyly is milder (partial syndactyly of the 2nd–3rd fingers as well as the 2nd–4th toes)	Imperforate anus, hydrocephalus, and radio-humeral synostosis	Obstructive sleep apnea, neurodevelopmental impairment, seizures, ocular complications. Type 1—better prognosis for intellectual outcome. Types 2 and 3—severe neurologic defects, death in childhood
Carpenter [15]	1 in 1 million live births	Autosomal recessive	RAB23 (RAS-associated protein)	Coronal, sagittal, and lambdoid craniosynostosis, which cause prominence of the metopic suture and brachycephaly	Cardiovascular anomalies, hypogonadism, omphalocele, polydactyly, brachydactyly, and clinodactyly	Craniosynostosis surgery with satisfactory esthetics of the craniofacial bones. Psychomotor delay, potential blindness, decreased visual acuity
Saethre–Chotzen [31]	20 in 1 million live births	Autosomal dominant	TWIST/FGFR2	Compared to Apert, patients have a towering forehead, low-set hairline, facial asymmetry, ptosis of the upper eyelids	Cutaneous syndactyly is usually partial and involves the 2nd–3rd fingers and/or the 3rd–4th toes	Sleep disorder breathing, developmental delays. Dental malocclusion, swallowing difficulties. Hearing loss, amblyopia
Muenke [32]	33.3 in 1 million live births	Autosomal dominant	FGFR3	Coronal synostosis or pan synostosis	Anterior plagiocephaly, temporal bossing, fusions of carpal and/or tarsal bones; brachydactyly, broad toes and thumbs, clinodactyly	Behavioral issues, seizures. Hearing loss, strabismus. Developmental delays, intellectual disabilities
Jackson-Weiss [33]	Unknown	Autosomal dominant	FGFR2 on chromosome 10q26	Craniosynostosis, mid-face hypoplasia, abnormally broad great toes, and/or malformation/fusion of bones from the feet.	-	Hearing impairment, normal intelligence, and a normal lifespan

FGFR—fibroblast growth factor receptor.

**Table 2:** An overview of the early prenatal diagnosis of Apert syndrome in newborns was all-time searched in the PubMed database.

Author/ Year	Maternal Age	GA at Diagnosis/Sex	Ultrasound/MRI Findings	Fetal Examination	FGFR2 Mutation	Outcome
Narayan 1991, [34]	29, mother with AS	19–20, F	The skull outline is very irregular and oval, “mitten” hands syndactyly.	Postmortem examination confirmed the features.	Not tested	Stillbirth, VD—34 wks
Ferreira 1999, [35]	33	20, M	Turribrachycephaly, acrocephaly, frontal bossing, short nose, bilateral 2nd-to-5th-finger syndactyly of the hand (‘mitten type’), abnormal toes.	The pathological examination confirmed the prenatal diagnosis.	S252W	TOP
Boog 1999, [36]	28	24, M	Turribrachycephaly, clover-leaf skull, midface hypoplasia, down slanting palpebral fissures, depressed nasal bridge, “mitten type” with 2nd-to-5th-finger total syndactyly of the hands, complete cutaneous fused toes with a broad big toe high forehead. MRI—brachycephaly and verticalization of the clivus with a flattened cranial base angle, hypertelorism.	The pathological examination confirmed the prenatal diagnosis.	Not tested	TOP
Lyu 2000, [37]	35, mother with AS	20, F	Frontal bossing, all cranial sutures widely separated, midface depression, syndactyly, fused toes.	The pathological examination confirmed the prenatal diagnosis, with both fontanels open.	Heterozygous 934C-G	TOP
Skidmore 2003, [38]	34	17.3/19 + 5, F	Clover-leaf skull, proptosis, VM, low-set ears, syndactyly of the hands, and broad first toes.	Turribrachycephaly, proptosis, a narrow and high arched palate, bilateral bony syndactyly, 2nd-to-5th-finger hands and feet, mitten-thumb, coarctation of the aorta.	755C → G, (S252W)	TOP
	33	21	Clover-leaf skull, VM, hypoplastic left heart.	Turribrachycephaly, hypertelorism, flat occiput, proptosis, beaked nose, midface hypoplasia, syndactyly, hypoplastic left ventricle, and hypo-plastic ascending aorta proximal to the ductus arteriosus.	755C → G, (S252W)	TOP
Hansen 2004, [39]	23	23, M	Abnormally shaped skull, syndactyly of the hands.	Craniosynostosis, craniofacial anomalies, bony and cutaneous syndactyly of the hands and feet.	Pro253Arg	VD at 32.5 wks
	26	21, F	Abnormally shaped skull, syndactyly, or an abnormal posturing of the hands.	N/A	Not tested	VD at 27 + 2 wks. Died at 4 m
Esser 2005, [40]	29	22	Widely open metopic suture with craniosynostosis, 2nd-to-4th-finger hands syndactyly.	Postmortem CT confirmed the prenatal findings. No autopsy was performed.	Heterozygous Pro253Arg (C758G)	TOP
Lam 2006, [41]	26	19	Strawberry head shape, craniosynostosis, ventricular septal defect, overriding of the aorta, syndactyly of the hands and feet.	Widely opened metopic suture, craniosynostosis, midface hypoplasia, bilateral syndactyly of the hands and feet. No autopsy was performed.	Heterozygous C1347G— Ser242Trp	TOP
Quintero-Rivera 2006, [42]	37	19, M	US—colpocephaly, ACC MRI—ACC, hypertelorism, oblong cranial shape.	Turricephaly, widely fontanelles and sagittal suture, fused toes, complex syndactyly, prominent downslanted eyes, hypertelorism, beaked nose, midface hypoplasia, depressed nasal bridge, mild tricuspid regurgitation. Postnatal 3D CT coronal craniosynostosis, wide midline calvarial defect, shallow orbits, midfacial hypoplasia.	S252W	CS at 39 wks
David 2007, [11]	N/A	21	Brachycephaly, VM, prominent forehead, proptosis, increased NT, absent nasal bone, abnormal DV, “mitten” hands, syndactyly of the hands and feet, arched foot, bilateral hydronephrosis.	The pathological examination confirmed the prenatal diagnosis.	S252W	TOP
	N/A	20 + 5	Long head, craniosynostosis, pro-ptosis, “mitten” hands, syndactyly of the hands and feet, small feet, abnormal position.	Syndactyly of the hands and feet, proptosis, and abnormal head shape. No autopsy was performed.	S252W	TOP
	N/A	22 + 5	Long head, flat occiput, mild VM, syndactyly of the hands, feet abnormal position, clitoromegaly.	The pathological examination confirmed the prenatal diagnosis.	P253R	TOP
Patel 2010, [22]	31	22 + 3	No significant brachycephaly, craniosynostosis, borderline VM, depressed nasal bridge, mild hypertelorism, cystic left orbit, or syndactyly of the hands and feet.	Postmortem 3D CT frontal bossing, wide metopic sutures with partial synostosis of coronal sutures, midface hypoplasia, hypertelorism, depressed nasal bridge. Autopsy—reasonable correlations of craniofacial anomalies.	Not tested	TOP
Respondek-Liberska 2010, [43]	31	22, F	22 wks—normal head shape, mild VM; 25 wks—colpocephaly, syndactyly of the hands and feet, mild hypertelorism; 28 wks—prominent forehead, craniosynostosis, midface hypoplasia, widely open metopic suture.	Typical facial appearance of AS, symmetrical, complex syndactyly of the hands 2nd-to-5th-fingers. The thumbs were not involved in the fusion. Feet syndactyly affected all toes.	S252W in exon 7	CS at term
Chen 2010, [44]	30	24, M	Midface hypoplasia, low-set ears, mild VM, mitten hand, 2nd-to-4th-finger syndactyly and abroad proximally deviated thumb.	Prominent midface hypoplasia, low-set ears, bilateral mitten syndactyly of the hands and feet, broad and proximally displaced thumbs, and big toes.	Heterozygous c.755 C>G, Ser252Trp (S252W)	TOP
Ludwig 2012, [45]	38	20 + 1, F	Turricephaly, frontal bossing, midface hypoplasia, low-set ears, hyperthelorism, and reduced facial movements.	Postmortem external examination—turribrachycephaly, prominent forehead, midface hypoplasia, proptosis, slightly down palpebral fissures, hypoplastic alae nasi, smooth palate cleft; hand—complete cutaneous syndactyly 2nd-to-4th-fingers II, III, IV with synonchia, Hitchhiker thumb; feet—broad halluces, complete cutaneous syndactyly toe 1st-to-4th-fingers, short V toe. Postmortem CT and MRI confirmed craniosynostosis, ACC, absence of the septum pellucidum, and complete fusion of the thalami.	Ser252Trp in exon 8	TOP
Ercoli 2014, [46]	30	23, M	Turricephaly, syndactyly, and omphalocele.	Turribrachycephaly, high arched palate, broad forehead, midface hypoplasia, hypertelorism, exophthalmos, syndactyly of hands and feet, bilateral cryptorchidism, omphalocele. Rx—craniosynostosis, synostosis between metacarpians and metatarsal bones.	Not tested	CS at 35 wks
Pi 2014, [47]	N/A	18/20/21/22 + 4, F	18 wks—cranial anomalies, syndactyly of the hands and feet; 20 wks—mild bilateral hydronephrosis, strawberry skull; 21 wks—craniosynostosis; 22 + 4 wks—craniosynostosis, low-set ears, frontal bulging, hypertelorism, syndactyly of the fingers of both hands.	Craniosynostosis, wide anterior fontanel, metopic suture dehiscence, synostosis of the lambdoid suture, ocular proptosis, low-set ears, medial palatal fissure, mild hypertelorism, the fingers and toes brachydactyly, syndactyly, 2–5 fingers and toes.	Heterozygous c.755C>G, p.Ser252Trp (NM 022970)	TOP
Giancotti 2014, [48]	37	21, M	US—irregular head shape, dolicocephaly, prominent forehead, flat occiput, bilateral mild VM, partial ACC, hypertelorism, midface hypoplasia, depressed nasal bridge, feet abnormal position, and syndactyly. MRI—excluded ACC.	Fetus macroscopic analysis: syndactyly of the hands (“spoon hands”) and feet, midface hypoplasia, prominent forehead.No postmortem autopsy.	Heterozygous c.758C>G (p.Pro253Arg)	TOP
Stark 2015, [49]	N/A	21	US—craniosynostosis, syndactyly, coarctation of the aorta. MRI—turribrachycephaly, CCD, temporal over-expansion/over-sulcation, transverse temporal clefts	The pathological examination confirmed the prenatal diagnosis.	c.755C>G (p.Ser252Trp)	N/A
Rubio 2016, [50]	N/A	22	US/MRI—calvarial indentation, hypertelorism, proptosis, syndactyly of the hands and feet.	N/A	NS	TOP
	N/A	21/30	US—calvarial indentation, “lamp-shade” calvarium, hypertelorism, syndactyly of the hands and feet, abnormally expanded lungs. MRI—plus proptosis, absent septum pellucidum.	N/A	NS	Survived
Wang 2017, [51]	27	23, F	Craniosynostosis, widely open metopic suture and anterior fontanel, midface hypoplasia, mitten hands, bony or cutaneous syndactyly of the hands and feet.	Fetal phenotype compatible with AS. No postmortem autopsy.	Not tested	TOP
Varlas 2017, [24]	32	21	Acrocephaly and brachycephaly, prominent forehead, nasal bone hypoplasia, exorbitism, flat midface, hypertelorism, low-set ears, cleft lip and palate, syndactyly of the hands and feet with lack of low extremities movements, vicious positions of the limbs.	The macroscopic examination confirmed the US diagnosis (craniosynostosis with acrocephaly and brachycephaly, flat midface, hypertelorism, cleft lip and palate, syndactyly of the hands and feet—the fusion of the bone and soft tissue components of all fingers).	Not tested	TOP
Quintas-Neves 2018, [52]	33	22 + 2	US—craniosynostosis, ocular proptosis, syndactyly of the hands and feet.MRI—turribrachycephaly, hypertelorism, over convolution, abnormal sulci in inferior and mesial temporal lobes.	N/A	c.755C>G (p.Ser252Trp)	TOP
Vieira 2019, [7]	33	22, F	US—prominent forehead, hypertelorism, proptosis, mitten hands. MRI—hypertelorism, turribrachycephaly, cranial dysmorphism, cortical malformation.	Craniosynostosis, hypertelorism, low-set ears, proptosis, bilateral syndactyly of the 2nd-to-5th fingers in hands and all fingers in the feet.	c.755C>G (p.Ser252Trp)	TOP
Shi 2020, [53]	32	23 + 5	Clover-leaf skull, syndactyly from the 2nd-to-5th finger.	Acrocephalosyndactyly and hypertelorism.	c.410C>G, chr10:123279677 (p.S137W)	TOP
Partoune 2021, [54]	39	23 + 5, M (twin)	Skull deformation, syndactyly—mitten hand. 27 wks—facial dysmorphism, small cerebellar vermis, syndactyly of the hands and feet, moderate VM, hypertelorism.	N/A	c.758C>G (p.Pro253Arg)	selective TOP at 33 + 4 wks
Zhang 2021, [55]	36	23, F	Unusual head shape, craniosynostosis, mild left VM, slight frontal bossing, proptosis, hypertelorism, syndactyly.	The pathological examination confirmed the prenatal diagnosis syndactyly of type VI (known as mitten type).	c.755C>G (p.S252W)	TOP
Tonni 2022, [56]	36	19, F	Turribrachicephaly, wide metopic suture, craniosynostosis, and syndactyly of the left hand.	Acrocephaly, broad forehead, mid-face hypoplasia, depressed nasal bridge, exophthalmos. The right hand—spade-shaped with thumb and index separated; the left hand—mitten-shaped with complete cutaneous syndactyly of fingers I-V, the feet—broad halluces and complete cutaneous syndactyly of all toes.	c.758C>Gp at 252 of the exon 8	TOP

GA—gestational age; AS—Apert syndrome; TOP—termination of pregnancy; VD—vaginal delivery; CS—cesarean section; ACC—agenesis of the corpus callosum; VM—ventriculomegaly; CCD—corpus callosum dysgenesis; DV—ductus venosus; wks—weeks; m—months; N/A—not available.

**Table 3:** Distribution of imaging (US, MRI) findings among Apert syndrome cases identified in the review.

Anomalies Findings	Ultrasound/MRI Findings *n* (%)
Cranial	
Abnormal head shape	31 (96.8%)
Suture morphology	15 (46.8%)
Ventriculomegaly	10 (31.2%)
ACC/DCC	2/1 (9.3%)
Facial	
Hypertelorism	14 (43.7%)
Proptosis	7 (21.8%)
Midface hypoplasia	8 (25%)
Prominent forehead	5 (15.6%)
Low-set ears	5 (15.6%)
Nasal deformity	5 (15.6%)
Limbs	
Syndactyly	28 (87.5%)
Abnormal toes	3 (9.3%)
Cardiovascular	3 (9.3%)
Renal	2 (6.2%)
Digestive	1 (3.1%)

ACC/DCC—agenesis/dysgenesis of corpus callosum.
